# Constructing an immune‐ and ferroptosis‐related lncRNA signature to predict the immune landscape of human bladder cancer

**DOI:** 10.1002/jcla.24389

**Published:** 2022-04-14

**Authors:** Xing Li, Libin Zhou, Tefei Lu, Lei Zhang, Yanjun Li, Jianting Xu, Min Yin, Huimin Long

**Affiliations:** ^1^ Department of Urology Ningbo Medical Center Lihuili Hospital Ningbo Zhejiang China

**Keywords:** bladder cancer, ferroptosis, immune infiltration, immunity, lncRNA, signature

## Abstract

**Background:**

LncRNAs play a variety of roles in the tumor microenvironment and cancer immune responses. Determining the significance of bladder cancer (BLCA)‐related genes to predict the prognostic and therapeutic response of BLCA is important.

**Methods:**

IrlncRNA/ frlncRNA pairs were determined using univariate analysis. The signature was constructed based on this pairs. Finally, analysis and internal validation were performed from several aspects.

**Results:**

We identified 60 immune‐ and ferroptosis‐related lncRNA pairs, among which 12 were included in the Cox proportional hazards model. Patients in low‐risk group survived for significantly longer. Survival and riskScore analyses showed that the low‐risk group had a significantly better clinical outcome. ROC curve analysis showed that AUC of OS values were more than 0.75 in the training set and the whole cohort. As assessed using Cox analysis, the riskScore was an independent prognostic predictor in the training, testing set and the whole cohort. The areas under the multi‐index ROC in the training set, the testing set, and the whole cohort were 0.777, 0.692, and 0.748, respectively. High‐risk group was positively associated with most of tumor‐infiltrating immune cells. High‐risk Scores correlated positively with high expression of CD274, but not with PD‐1. Low riskScores correlated positively with high expression levels of the genes ERBB2 and nectin‐4. High‐risk Score was associated with a lower IC50 value for Docetaxel, cisplatin, and Pazopanib, while there was an opposite result for metformin.

**Conclusions:**

The signature constructed by pairing irlncRNAs and frlncRNAs showed a notable clinical predictive value.

## Graphical abstract

The Figure 1 showed workflow of the present study, including data acquisition, filtering, model construction and verification process.

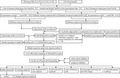

## 1 INTRODUCTION

Bladder cancer (BLCA) is the 10th most commonly diagnosed cancer worldwide, with approximately 573,000 new cases and 213,000 deaths in 2020 and is also the fourth most prevalent cancer in men.^1^, ^2^ Nonmuscle‐invasive bladder cancer (NMIBC) and a part of muscle‐invasive bladder cancer (MIBC) comprise non‐metastatic bladder cancer. About a quarter of patients with BLCA suffer from MIBC or metastatic foci.^3^, ^4^ Moreover, the recurrence rate of BLCA is high, and after surgery, approximately 50% of patients suffer relapse and develop metastases.^5^, ^6^ Patients with BLCA have benefitted markedly from adjuvant chemotherapy and new immune checkpoint inhibitors (ICIs).^7^, ^8^, ^9^, ^10^, ^11^, ^12^ However, there is still a considerable proportion of patients who do not respond to immunotherapy at all stages of BLCA because of tumor immune evasion.^13^

Lipid peroxidation‐mediated and iron‐dependent cell death is termed ferroptosis, which differs from autophagy, necrosis, and apoptosis.^14^ Ferroptosis is involved in many diseases, including cancer.^15^, ^16^ In a recent study, researchers found that CD8+ T cells participate in the regulation of tumor ferroptosis during cancer immunotherapy.^17^ Another study showed that immunotherapy‐promoted tumor ferroptosis and the effects of combination immunotherapy were enhanced using iron oxide‐loaded nanovaccines (IONVs).^18^ For bladder cancer, recently studies indicate that PGE2 metabolism affects variety of immune cells function and eventually lead to immune evasion.^19^ In addition, the release of PGE2 could induct ferroptosis in cancer cells.^20^ Altogether, these data indicate that ferroptosis is closely related to the antitumor immunity of bladder cancer.

Long non‐coding RNAs (lncRNAs) are non‐coding transcripts with a length >200 nucleotides.^21^ LncRNAs comprise almost 80% of the human transcriptome and play a key role in post‐transcriptional regulatory processes related to mRNA translation, stability, or splicing.^22^ A recent study also showed that lncRNAs are critical to regulate genes encoding proteins that participate in cancer immunity.^23^ LncRNAs are also important for immune‐cell infiltration into tumors.^24^

Previous studies have focused on signatures of immune‐ or ferroptosis‐related lncRNAs, or on immune‐ and ferroptosis‐related mRNAs.^25^, ^26^, ^27^, ^28^ Although these studies demonstrated excellent value for the prediction and prognosis for cancer diagnosis, evaluation, and treatment, the roles of immune‐ and ferroptosis‐related lncRNA pairs are rarely studied. Therefore, the present study aimed to employ a novel algorithm to develop an immune‐related lncRNA (irlncRNA) and ferroptosis‐related lncRNA (frlncRNA) signature that does not rely on specific lncRNA expression levels and expected that it could more accurately predict the prognosis of patients and the response to immunotherapy in patients with bladder cancer. Its predictive value was estimated for patients with BLCA, and its chemotherapy efficacy, diagnostic effectiveness, immune checkpoint‐related genes, and antibody‐drug conjugate‐related genes were determined.

## 2 MATERIALS AND METHODS

### 2.1 Transcriptome data retrieval, analysis of differential expression, and intersection analysis

We downloaded the human BLCA transcriptome profile (RNA sequencing data), harmonized as fragments per kilobase of transcript per million mapped reads (FPKM), from The Cancer Genome Atlas (TCGA: https://tcga‐data.nci.nih.gov/tcga/) for follow‐up analysis. To distinguish the lncRNAs from mRNAs, we also downloaded gene transfer format (GTF) files from Ensembl (http://asia.ensembl.org). Then, we downloaded a list containing recognized immune‐related genes (ir‐genes) from the ImmPort database (http://www.immport.org), which was used in co‐expression analysis to identify irlncRNAs. Similarly, co‐expression analysis using a list of 288 recognized ferroptosis‐related genes (fr‐genes), downloaded from the FerrDb dataset (http://www.zhounan.org/ferrdb/), was used to identify frlncRNAs. A correlation coefficient of more than 0.4 and a *p*‐value <0.01 were used as the criteria to identify irlncRNAs and frlncRNAs. To identify the differentially expressed DEirlncRNAs and DEfrlncRNAs, the R package limma was used, with thresholds of log fold change (FC) >2 and a false discovery rate (FDR) <0.05. Venny 2.1 (https://bioinfogp.cnb.csic.es/tools/venny/index.html) was used to complete the gene intersection analysis.

### 2.2 DE‐IFRLs pairing

The DE‐IFRLs were cyclically matched, and a 0‐or‐1 matrix was constructed supposing that C was a DE‐IFRLs pair composed of lncRNA A and lncRNA B; If lncRNA A has a higher expression than lncRNA B, C was defined as 1; otherwise, C was defined as 0. Then, we further screened the established 0‐or‐1 matrix. The relationship between pairs was not considered if the expression level of an lncRNA pair was 0 or 1. The reason was that pairs could not predict patient survival outcome properly unless they had a certain rank. It was considered an effective matching if the number of lncRNA pairs with an expression level of 0 or 1 accounted for more than 20% of the total pairs.

### 2.3 Patients’ clinical data

The clinical data for patients with BLCA was retrieved from the BLCA project of the TCGA. Effective data were obtained by excluding repeated data and data with a follow‐up of less than 30 days.

### 2.4 Developing a risk model to evaluate the riskscore

First, we performed single factor analysis. Second, the patients were randomly divided into the training and testing sets at a ratio of 3:2 (237, 158) and we used least absolute shrinkage and selection operator (LASSO) regression in the training set, with a *p*‐value of 0.05 and 10‐fold cross validation. 1000 cycles of LASSO regression were run, and 1000 simulations were set for each cycle. Third, we recorded the frequency of each pair in the LASSO regression model. Fourth, Cox proportional hazard regression analysis was performed for pairs with a frequency >100, which were also used to construct the model. The following formula was then used to evaluate the riskScore for the constructed risk model for all the clinical cases:

$$\text{RiskScore}=\sum_{i=1}^{n}\text{Coef}(i)*E\left(i\right)$$

Clef (i) and E(i) represent the regression coefficient of the multivariate Cox analysis for the DEfirlncRNA pairs and the expression value of each DEfirlncRNA pair, respectively. Finally, patients in the training set were classified into low‐ and high‐risk groups according to the median riskScore. The testing set and the whole cohort were also grouped using the same the median riskScore.

### 2.5 Risk model validation

Univariate and multivariate Cox analysis were utilized to validate the lncRNA pairs in the model. Then, Kaplan–Meier analysis, time‐dependent receiver‐operating characteristic (ROC) analysis, riskScore analysis, and survival outcome analysis was used to demonstrate the survival difference of patients in the low‐ or high‐risk groups. Next, R tools were used to visualize the results above by using the R packages of survival, survminer, and survivalROC.

Finally, to verify that the model could be used as an independent clinical prognostic predictor, multi‐index ROC analysis, and univariate and multivariate Cox regression analyses were performed between the riskScore and the clinicopathological characteristics. The results are presented using a forest plot and ROC curve. These analyses used the R package survival.

### 2.6 Tumor‐infiltrating immune cell, immune checkpoint‐related genes, and antibody‐drug conjugate‐related genes

To analyze the relationship between the riskScore and immune‐cell characteristics, the immune infiltration status of the BLCA samples from the TCGA project were evaluated using CIBERSORT,^29^, ^30^ MCPcounter,^31^ QUANTISEQ,^32^, ^33^ EPIC,^34^ TIMER,^35^, ^36^ XCELL,^37^, ^38^ and CIBERSORT‐ABS.^39^ The relationship between the riskScore values and the immune infiltrated cells was determined using Spearman correlation analysis and a lollipop diagram was used to display the result. Then, the Wilcoxon signed‐rank test was used to analyze the differences in immune checkpoint‐ and antibody‐drug conjugate‐related genes among the different groups; the results of which are shown in a box chart. A *p*‐value <0.05 was set as the significance threshold. The R limma, scales, ggtext, ggplot2, and ggpubr packages were used to perform these procedures.

### 2.7 Determining the model's significance in clinical treatment

The BLCA dataset was mined for the half‐maximal inhibitory concentration (IC50) values of commonly used chemotherapeutic drugs, with the aim of assessing the clinical significance of the model for BLCA treatment. The commonly used drugs comprised cisplatin, pazopanib, and docetaxel. Recent studies have suggested the therapeutic efficacy of metformin in certain cancers; therefore, we also explored the relationship between metformin and the model. The Wilcoxon signed‐rank test was used to compare the differences in IC50 among the groups. The analysis was carried out using pRRophetic and ggplot2 of R and the results are presented using box plots.

## 3 RESULTS

### 3.1 Identification of differentially expressed irlncRNAs (DEirlncRNAs), frlncRNAs (DEfrlncRNAs) and determining the intersection of the two sets of DElncRNAs

**FIGURE 1:**
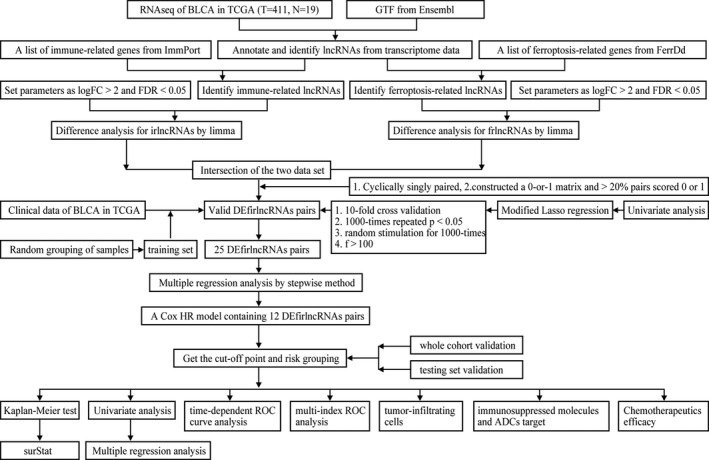
Workflow of the present study

**FIGURE 2:**
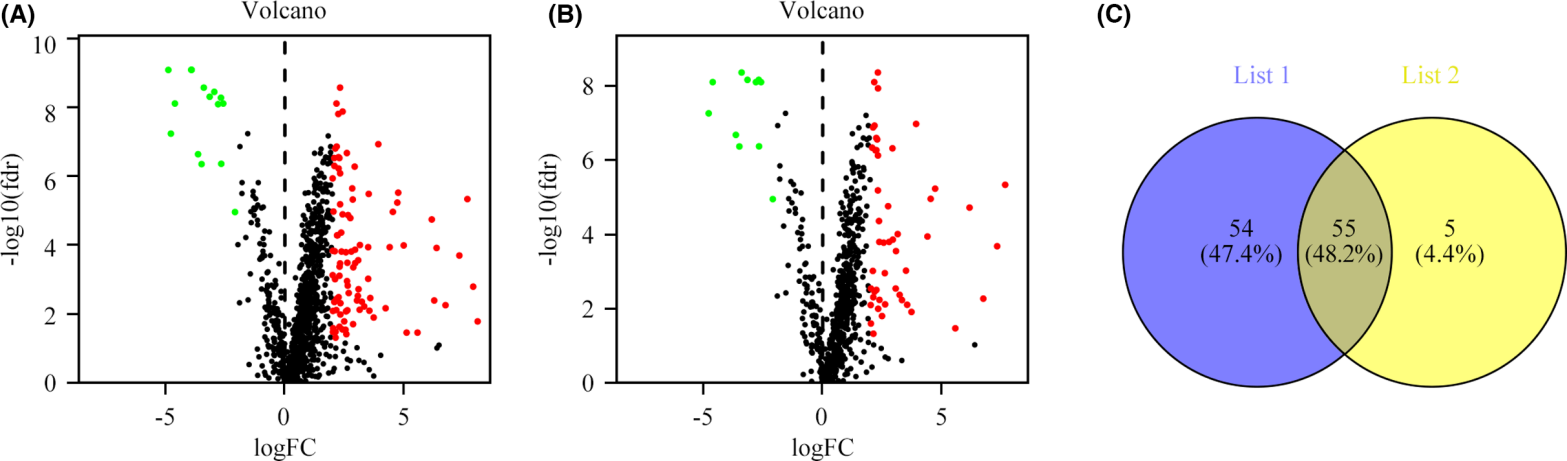
Identification of differentially expressed immune‐related lncRNAs (DEirlncRNAs) and ferroptosisrelated lncRNAs (DEfrlncRNAs) from TCGA data and Ensembl‐based annotation. (A) DEirlncRNAs shown on a volcano plot (B) DEfrlncRNAs shown on a volcano plot. (C) Intersection of the two sets of DElncRNAs

Figure 1 shows the flowchart of the study. First, the BLCA transcriptome profile data were downloaded from the TCGA database, comprising 19 normal samples and 411 tumor samples. The ir‐genes list was obtained from the IMMPORT Shared Data. Then, a list of 288 recognized ferroptosis‐related genes (fr‐genes) were downloaded from the FerrDd dataset. The data were then annotated according to the GTF files and used for co‐expression analysis between the lncRNAs and the ir‐genes. This analysis identified 1270 irlncRNAs, 109 of which were found to be differentially expressed (DEirlncRNAs), with 94 being upregulated and 15 being downregulated (Figure 2A). The same analysis was used for the fr‐genes and lncRNAs, which identified 60 of them as DEfrlncRNAs, with 49 being upregulated and 11 being downregulated (Figure 2B). The intersection of the two DElncRNAs datasets is shown in Figure 2C.

### 3.2 Establishing IFRL pairs and the risk assessment model

**FIGURE 3:**
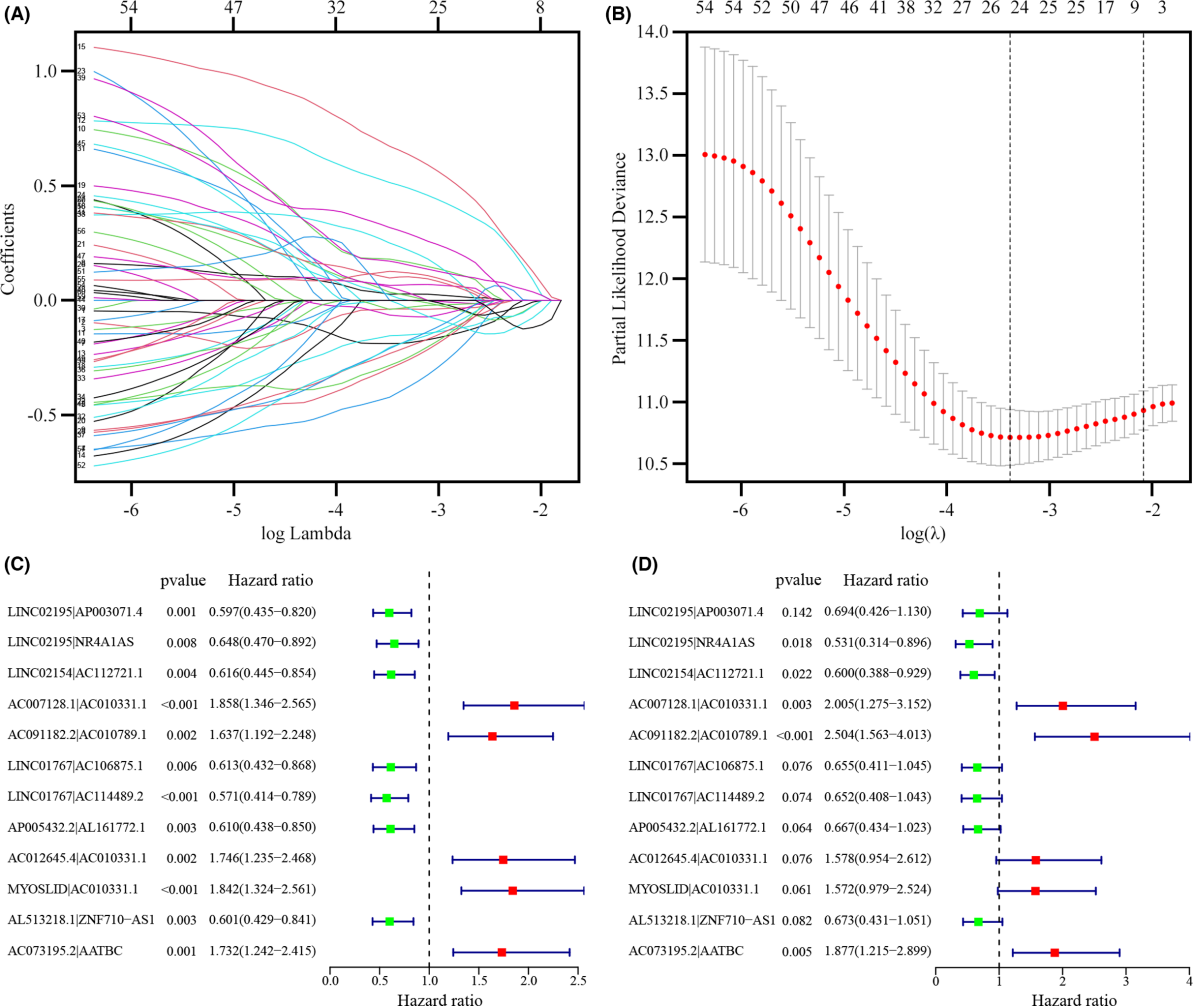
Establishment of the Risk Assessment Model using DE‐IFRL Pairs (A, B) The 60 pairs of prognostic lncRNAs were used to construct a LASSO Cox regression model, and the partial likelihood deviance incorporating 10‐fold cross‐validation was used to derive the tuning parameter (λ). In the plot, the vertical black line indicates an optimal log λ. (C, D) Results of (C) univariate and (D) multivariate Cox regression analyses of lncRNA pairs involved in the model

An iteration loop and a 0‐or‐1 matrix were used to screen 55 IFRLs, among which 1220 valid IFRL pairs were identified. A single factor test extracted 60 IFRL pairs, followed by modified LASSO regression analysis (Figure 3A,B). The stepwise method resulted in twelve of these IFRL pairs (LINC02195|AP003071.4, LINC02195|NR4A1AS, LINC02154|AC112721.1, AC007128.1|AC010331.1, AC091182.2|AC010789.1, LINC01767|AC106875.1, LINC01767|AC114489.2, AP005432.2|AL161772.1, AC012645.4|AC010331.1, MYOSLID|AC010331.1, AL513218.1|ZNF710‐AS1, AC073195.2|AATBC) being included in a Cox proportional hazards model (Figure 3C,D).

### 3.3 Clinical evaluation using the risk assessment model

**FIGURE 4:**
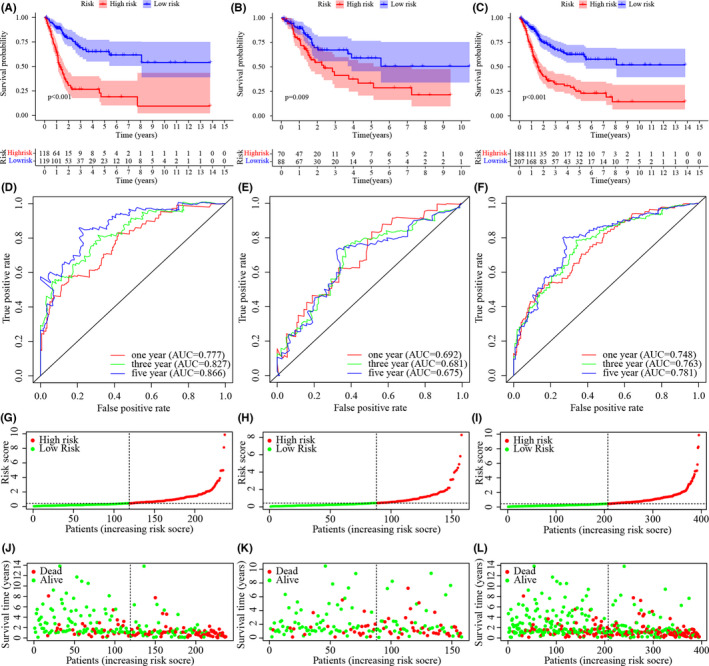
Validation of the Risk Assessment Model (A, B, C) Kaplan–Meier tests in training set (A), the testing set (B), and the whole cohort (C). (D, E, F) time‐dependent ROC analysis of risk scores based on 1‐, 3‐, and 5‐year OS in the raining set (D), the testing set (E), and the whole cohort (F). (G‐L) Risk scores of each case and Survival outcome of each case in the training set (G, J), testing set (H, K), and the whole cohort (I, L)

**FIGURE 5:**
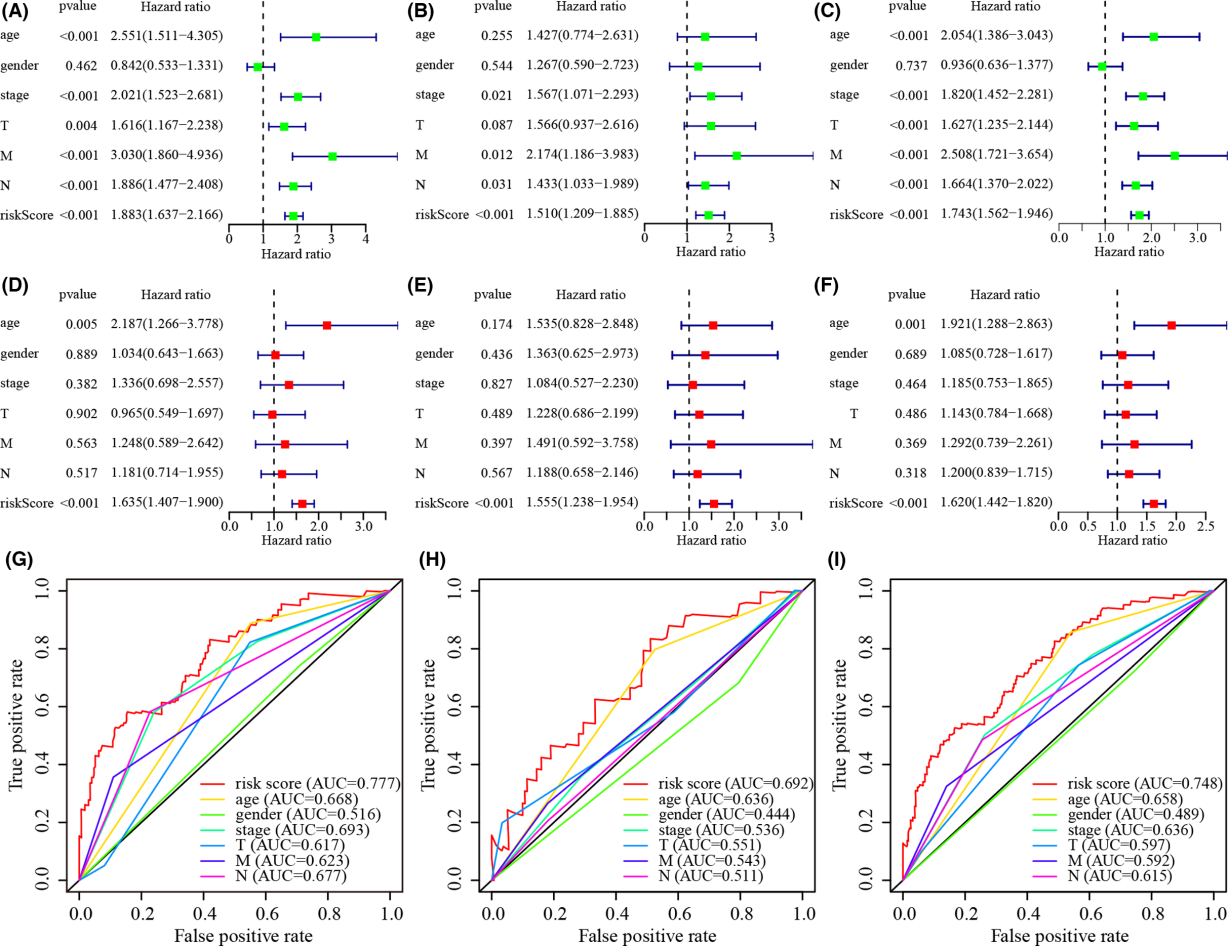
Use of the risk assessment model for clinical evaluation. (A‐F) Results of Univariate Cox and Multivariate Cox analysis showing the relationship of the risk score and clinical variables including age, sex, and TNM stage to overall survival (OS) in the training set (A, D), the testing set (B, E), and the whole cohort (C, F); (G‐I) multi‐index ROC curve analysis of the signature demonstrated that the areas under the curves in the training set, the testing set, and the whole cohort were 0.777, 0.692, and 0.748, respectively

According to Kaplan–Meier analysis, the patients in the low‐risk group survived for significantly longer than the patients in the high‐risk group (Figure 4A–C). The riskScores and survival of these cases are shown in Figure 4G–I and 4J–L. The results showed that the low‐risk group had a significantly better clinical outcome than the high‐risk group. Next, in the time‐dependent ROC curve analysis, the area under the curve (AUC) of OS for 1, 3, and 5 years was 0.777, 0.827, and 0.866 in the training group (Figure 4D); 0.692, 0.681, and 0.675 (Figure 4E) in the testing group; and 0.748, 0.763, and 0.781 (Figure 4F) in the whole cohort, respectively, which further confirmed the validity of our results. Finally, univariate (Figure 5A–C) and multivariate Cox regression analyses in both the training and validation groups showed that age and riskScore were independent prognostic predictors of OS in the training set and the whole cohort (Figure 5D,5F), but only the riskScore was an independent prognostic predictor in the testing set (Figure 5E). Multi‐index ROC analysis was performed for further validation (Figure 5G–I). The areas under the curves in the training set, the testing set, and the whole cohort were 0.777, 0.692, and 0.748, respectively.

### 3.4 Analyses of tumor‐infiltrating immune cells, immunosuppressive molecules, and ADC targets using the risk assessment model

**FIGURE 6:**
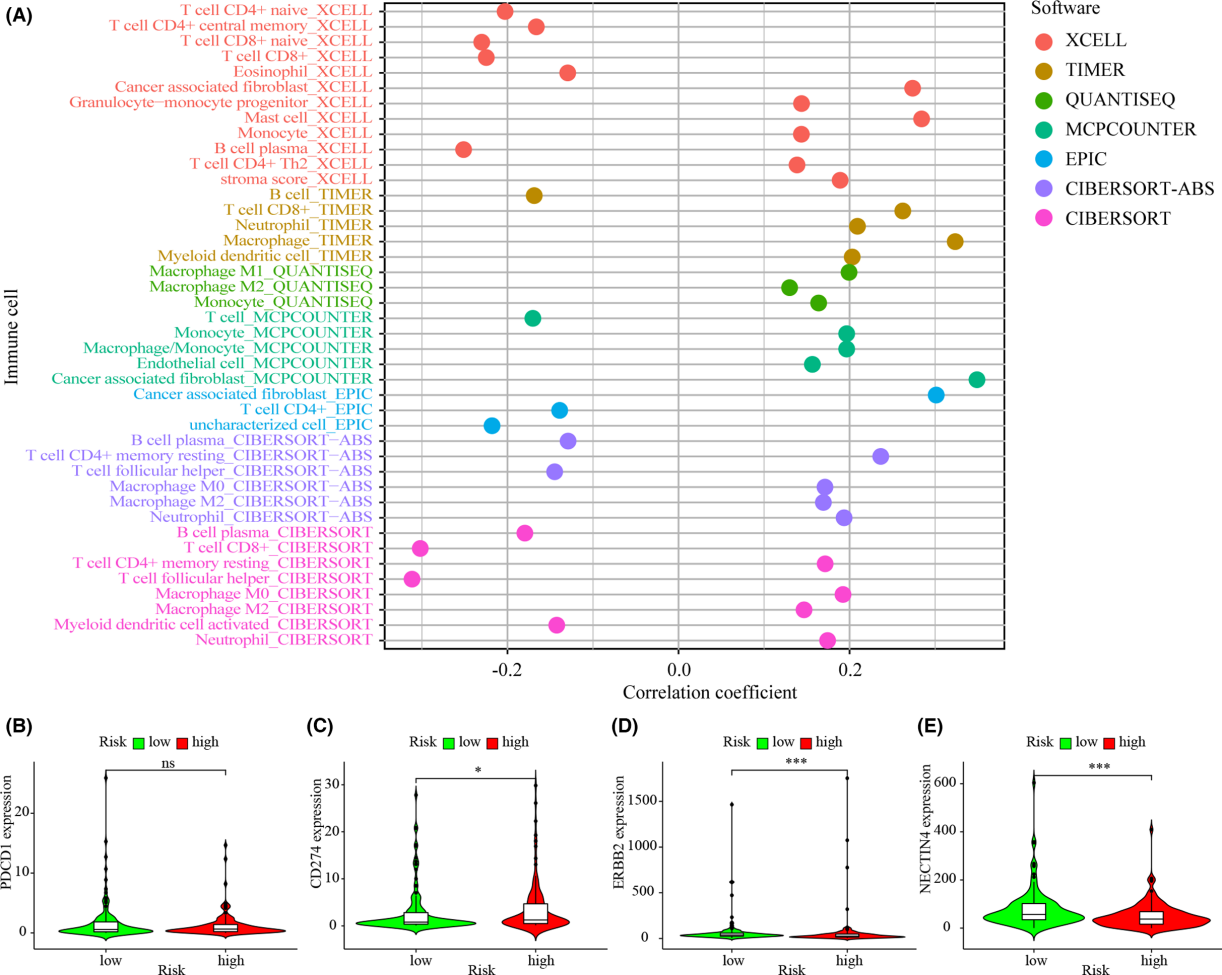
Use of the risk assessment model to estimate tumor‐infiltrating cells, immunosuppressed molecules, and ADC targets. (A) Spearman correlation analysis showing that tumor‐infiltrating immune cells such as neutrophils, monocytes, fibroblasts, and macrophages, were associated positively with patients in the high‐risk group, whereas these patients were associated negatively associated with CD4+ T cells and fibroblasts. (B, C) The upregulated level of CD274 correlated positively with high‐risk scores (C), whereas the expression level of PDCD1 was not different among the groups (B). (D, E) Upregulated level of ERBB2 (D) and nectin‐4 (E) correlated positively with low‐risk scores. *p < 0.05; **p < 0.01; ***p < 0.001

Immune‐related genes and lncRNAs are interrelated; therefore, we explored the association between the model and the tumor immune microenvironment. The “infiltration_estimation_for_tcga.csv” data file was downloaded from Timer database. We then performed correlation analysis utilizing Spearman analysis. The results showed that the high‐risk group was positively associated with tumor‐infiltrating immune cells, including monocytes, fibroblasts, and macrophages, but negatively related to CD4+ T cells and CD8+ T cells (Figure 6A). ICIs comprise very important treatments for patients with BLCA in clinical practice; therefore, we assessed whether the model was associated with ICI‐related biomarkers. The results showed that high‐risk Scores correlated positively with high expression levels of the gene encoding CD274 (also known as PD‐L1) (*p* < 0.05, Figure 6B); however, there was no significant relation between the riskScores and the expression levels of PDCD1 (also known as PD‐1) (Figure 6C). Antibody‐drug conjugates represent a class of emerging therapeutics; therefore, we also investigated whether the model associated with relevant biomarkers. The results showed that low riskScores correlated positively with high expression levels of the gene encoding Erb‐B2 receptor tyrosine kinase 2 (ERBB2, also known as HER‐2) (*p* < 0.001, Figure 6D) and nectin‐4 (*p* < 0.001, Figure 6E).

### 3.5 Analysis of the correlation between the risk model and chemotherapeutics

**FIGURE 7:**
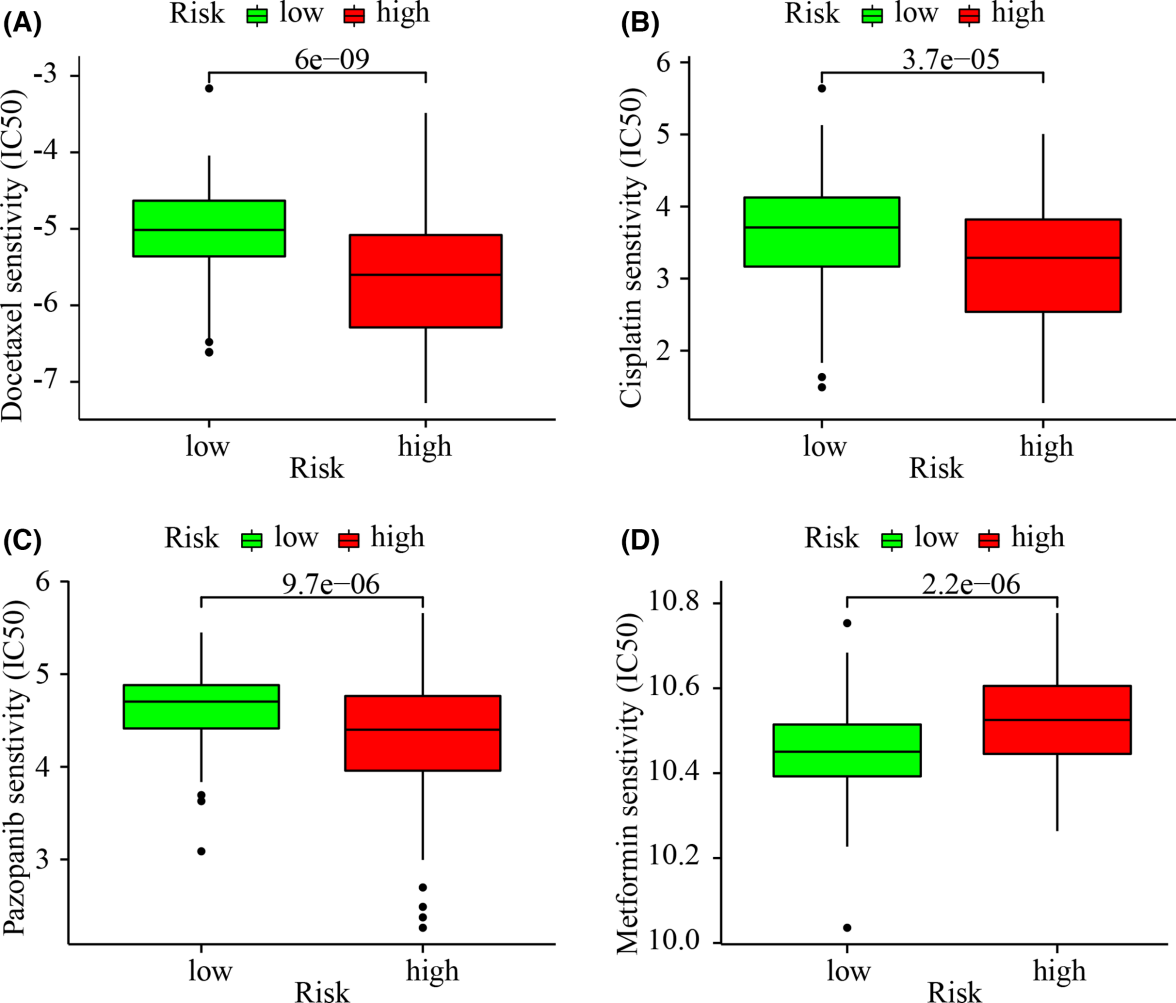
The model could function to predict chemosensitivity. High‐risk scores were associated with lower IC50 values for targeted drugs (e.g., Pazopanib) (C) and chemotherapeutics (e.g., cisplatin and doxorubicin) (A, B), but were associated with higher IC50 scores for Metformin (D)

We attempted to discover the association between the riskScore and the efficacy of commonly used chemotherapeutic and targeted drugs. The results showed that a high‐risk Score was associated with a lower IC50 value for Docetaxel (*p* < 0.001), cisplatin (*p* < 0.001), and Pazopanib (*p* < 0.001), which suggested that the developed model could be used to predict chemotherapeutic drug sensitivity (Figure 7A–C). In the case of metformin, the IC50 in the low‐risk group was lower than that in the high‐risk group (*p* < 0.001, Figure 7D).

## 4 DISCUSSION

Immunotherapy of a considerable proportion of patients with BLCA is limited by immune invasion. In fact, recent studies have shown that, *in vitro*, macrophages effectively engulf ferroptotic cancer cells, supporting the existence of "find me" and "eat me" signals.^40^, ^41^ The critical steps in the interaction between ferroptotic cancer cells and the immune system are phagocytosis, migration, maturation, antigen processing, and cross‐presentation by DCs.^42^ Previous research indicated that PGE2 metabolism in the bladder cancer promoted the formation of immunosuppressive tumor‐supporting microenvironment and could induct ferroptosis in cancer cells.^19^, ^20^ Therefore, we speculate that ferroptosis that happened in the tumor microenvironment has a negative impact on the antitumor immunity of bladder cancer patients and the model constructed by ferroptosis/immune lncRNA would have better prediction ability for the prognosis of patients and the response of medicine.

Recently, to assess the prognosis of patients with tumors, researchers have focused on establishing signatures based on coding genes, non‐coding RNAs, and non‐coding RNA pairs,^25^, ^26^, ^27^, ^28^, ^43^, ^44^, ^45^, ^46^, ^47^, ^48^, ^49^, ^50^, ^51^, ^52^, ^53^, ^54^ most of which are based on the quantification of gene expression levels. Moreover, most of them were associated with either immune‐related or ferroptosis‐related RNAs. Herein, we used a strategy of immune‐related and ferroptosis‐related lncRNA pairing to construct a valuable model that does not depend on their detailed expression level. The results are basically consistent with our expectations above.

First, TCGA raw data were used to identify DEirlncRNAs and DEfrlncRNAs. After determining the intersection of the two DElncRNAs sets, an improved method comprising a 0‐or‐1 matrix and cyclical single pairing was utilized to validate the lncRNA pairs. Second, univariate analysis together with modified LASSO regression (including random simulation, multiple repeats, and cross validation) were used to determine the intersecting pairs. Third, the obtained formula was used to evaluate the riskScores, and cases were divided into low‐and high‐risk groups according to the median riskScore. Subsequent reassessment and validation of the survival outcome and analysis of clinicopathological characteristics showed that the developed model worked well.

It is reported that the response to anti‐checkpoint blockades is affected by the intertumoral infiltration of immune cells. To determine the relationship between tumor‐infiltrating immune cells and risk scores, seven methods were used, including TIMER, CIBERSORT, XCELL, QUANTISEQ, MCPcounter, EPIC, and CIBERSORT‐ABS. Comparisons among the algorithms are rarely performed because of their various limitations and complexities. Our results showed that the high‐risk group was associated with certain tumor‐infiltrating immune cells, including neutrophils, myeloid dendritic cells, macrophages, and monocytes.

In recent years, the role of ferroptosis in immunotherapy has aroused much interest. In one study of immunotherapy‐associated cytokines, the authors observed that inducers of ferroptosis had impact on the differentiation of melanoma cells and affected the antitumor efficacy of immunotherapy.^55^ Certain physiological processes induced by ferroptosis could, to some extent, activate innate immunity.^56^ Wu's study found that patients with bladder cancer in the high‐risk group of the irlncRNA signature had high expression of MSH6 (MutS homolog 6) and MHL1 (MutL homolog 1), a low TMB, and low expression of programmed cell death 1 (PD‐1) and programmed cell death 1 ligand 1 (PD‐L1).^27^ Research on hepatocellular carcinoma showed that the signature correlated with immune checkpoint‐related biomarkers such as CTLA4 and HAVCR2, but not PD‐1 and LAG3.^57^ Our study identified positive associations between the riskScore and PD‐L1 gene expression. This suggested that the combined use of ferroptosis‐related drugs with ICIs in the high‐risk group would benefit patients. Antibody‐drug conjugates (ADCs) represent a new therapeutic modality in urothelial cancer^58^ . ADCs targeting nectin‐4 were approved to treat bladder cancer in 2019 by the FDA^58^ and an ADC targeting HER‐2 was approved in 2021 by the CSCO Guideline depending on the C005 research study^59^ . Recent research showed that the expression of Nectin‐4 and HER‐2 are related to ferroptosis^60^, ^61^, ^62^ . Other studies have explored the design of PD‐L1 ADCs^63^, ^64^ . Our study indicated that the expression of Nectin‐4 and HER‐2 was increased significantly in the low‐risk group compared with that in the high‐risk group, which showed the ability of the model to predict the sensitivity of ADCs.

However, the present research had several limitations. First, the raw dataset, which was simply downloaded from the TCGA, was comparatively insufficient and we have only performed internal validation. Hence, external validation and additional prospective investigations are needed to validate the predictive power of our model. Second, we did not retrieve datasets for the other information, such as clinicopathological characteristics, lncRNA expression levels, and survival outcomes at the same time. Third, although the signature was constructed using lncRNA pairs, fresh samples and prospective experimental research are required to validate these lncRNAs. Finally, the biological functions of the lncRNAs making up the prognostic signature need to be explored in detail in bladder cancer.

## 5 CONCLUSION

In the present work, we constructed an irlncRNAs and frlncRNAs signature that was independent of the expression levels of lncRNAs. The signature could be used for prognosis prediction in patients with BLCA and could facilitate decisions regarding whether a patient might respond to BLCA immunotherapy and ADCs targeting Nectin‐4 and HER‐2.

## CONFLICT OF INTERESTS

The authors have declared that no competing interest exists.

## AUTHOR CONTRIBUTIONS

Xing Li and Jianting Xu conceived the study; Libin Zhou and Tefei Lu performed the R language analysis; Lei Zhang and Yanjun Li contributed significantly to the analysis and manuscript preparation; Xing Li performed the data analyses and wrote the manuscript; Huimin Long and Min Yin helped perform the analysis and provided constructive discussions.

## Data Availability

The datasets presented in the study are included in the article, further inquiries can be directed to the corresponding authors.
